# Variations in Rainfall Affect the Responses of Foliar Chemical Properties of *Cunninghamia lanceolata* Seedlings to Soil Warming

**DOI:** 10.3389/fpls.2021.705861

**Published:** 2021-07-30

**Authors:** Qiufang Zhang, Dawei Luo, Liuming Yang, Jinsheng Xie, Zhijie Yang, Jiacong Zhou, Xiaojie Li, Decheng Xiong, Yuehmin Chen, Yusheng Yang

**Affiliations:** ^1^College of Geographical Science, Fujian Normal University, Fuzhou, China; ^2^State Key Laboratory of Subtropical Mountain Ecology (Funded by Ministry of Science and Technology and Fujian Province), Fujian Normal University, Fuzhou, China; ^3^College of Urban and Environmental Sciences, Peking University, Beijing, China; ^4^Department of Renewable Resources, Faculty of Agricultural, Life and Environmental Sciences, University of Alberta, Edmonton, AB, Canada

**Keywords:** warming, subtropics, stoichiometry, nutrient limitation, growth

## Abstract

Climate warming is becoming an increasingly serious threat. Understanding plant stoichiometry changes under climate warming is crucial for predicting the effects of future warming on terrestrial ecosystem productivity. Nevertheless, how plant stoichiometry responds to warming when interannual rainfall variation is considered, remains poorly understood. We performed a field soil warming experiment (+5°C) using buried heating cables in subtropical areas of China from 2015 to 2018. Stoichiometric patterns of foliar C:N:P:K:Ca:Mg, non-structural carbohydrate, and stable isotope of *Cunninghamia lanceolata* seedlings were studied. Our results showed that soil warming decreased foliar P and K concentrations, C:Ca, P:Ca, and P:Mg ratios. However, soil warming increased foliar Ca concentration, δ^15^N value, C:P and N:P ratios. The response ratios of foliar N, C:N, and δ^15^N to soil warming were correlated with rainfall. Our findings indicate that there was non-homeostasis of N and C:N under warming conditions. Three possible reasons for this result are considered and include interannual variations in rainfall, increased loss of N, and N limitation in leaves. Piecewise structural equation models showed that stoichiometric non-homeostasis indirectly affected the growth of *C. lanceolata* seedlings in response to soil warming. Consequently, the growth of *C. lanceolata* seedlings remained unchanged under the warming treatment. Taken together, our results advance the understanding of how altered foliar stoichiometry relates to changes in plant growth in response to climate warming. Our results emphasize the importance of rainfall variations for modulating the responses of plant chemical properties to warming. This study provides a useful method for predicting the effects of climate warming on economically important timber species.

## Introduction

The global average temperature has reportedly increased by 0.85°C compared with the pre-industrial levels (IPCC, 2018), and this has raised widespread attention. Current research shows that increasing temperatures have positive (Day et al., 2008; Wu et al., 2020), neutral (Mao et al., 2016; Peng et al., 2020), or negative (Herguido et al., 2016; Taccoen et al., 2021) effects on plant growth. These discrepancies may be related to the differences in soil nutrient availability and plant nutrient requirements.

Plant carbon: nitrogen: phosphorus (C:N:P) stoichiometric variance can be used to predict the relative status of plant nutrient requirements from subcellular to ecosystem scales (Sterner and Elser, 2002). Although the responses of plant C:N:P stoichiometry to warming have been extensively reported at global (Reich and Oleksyn, 2004), regional (Zhang et al., 2012), and local (Zhang et al., 2017) scales, the results are highly variable. Different methods, including greenhouses (Day et al., 2008), open top chambers (Nybakken et al., 2011), soil heating cables (Zhang et al., 2019), reflectors (Sardans et al., 2008a), and infrared heaters (Dijkstra et al., 2012), may have contributed to the differences in results (Yue et al., 2017). Many other factors, such as rainfall, developmental stage, and resource supply might also affect the response of plant stoichiometry to warming (Han et al., 2011). One-time sampling only provides a “snapshot” of the plant's response to warming, and the details of seasonal and interannual variations in these responses warrant further investigation (Ren et al., 2021).

Stoichiometric homeostasis suggests that, plant growth rate will be maximized if the stoichiometry of resource supply matches that of plant demand (Sterner and Elser, 2002). In contrast, if the stoichiometry of resource supply mismatches that of plant demand (i.e., stoichiometric non-homeostasis), plant growth rate will be limited by particular nutrients. Climate warming may trigger numerous molecular responses in plants. Yu et al. (2015) reported that primary producers exhibited strong stoichiometric homeostasis, and each species had its own stoichiometric ratio and survival strategy. Chen et al. (2020) conducted warming experiments in an alpine meadow grassland and found that different species had different responses to warming, but overall, warming significantly advanced plant phenology. Plants growing in low-latitude regions have a narrower temperature tolerance range than those growing at high-latitudes (Cavaleri et al., 2015). A study conducted in southern China suggested that warming significantly increases easily available and moderately available soil P (Yang et al., 2019). This combination of factors is expected to improve forest productivity. However, the aboveground height of *Cunninghamia lanceolata* in the same location has no significant change to soil warming (Xiong et al., 2018). It was reported that the growth of *C. lanceolata* seedlings was limited by N (Zhang et al., 2019). The interaction between N and P metabolism may be important for the regulation of plant growth and maintenance of nutrient homeostasis in changing environments (Sterner and Elser, 2002; Wang et al., 2018).

In addition to C, N, and P, recent studies have taken into account additional mineral elements, such as potassium (K), calcium (Ca), and magnesium (Mg), which would improve our understanding of how warming affects basic biological functions such as growth, stress responses, and defense mechanisms (Prieto and Querejeta, 2020; Gao et al., 2021; Sardans et al., 2021). For example, it was reported that warming increased Ca concentration in *Erica multiflora* and *Lotus dorycnium* by 42 and 38%, respectively, which helped to improve the water-use efficiency of plants (Sardans et al., 2008b). δ^13^C in leaves can systematically reflect the response of photosynthetic gas exchange and transpiration to environmental changes (Huang et al., 2015; Belmecheri et al., 2021). Previous studies have reported that the value of δ^13^C showed a more consistent seasonal signal for climate change across sites than ring width (Maxwell et al., 2020). In addition, non-structural carbohydrate is the most important mobile C pool and the material basis for growth and metabolism in organisms; they mainly include soluble sugar and starch (Du et al., 2020; Herrera-Ramirez et al., 2020). In well-irrigated conditions, *Panicum maximum* has been reported to maintain stoichiometric homeostasis in a warming climate, and this was related to the distribution of sugars among different organs (Viciedo et al., 2019). Although the importance of these proxies is known, whether warming will increase K, Ca, Mg, and non-structural carbohydrate concentrations and water-use efficiency has not been tested in low-latitude forests.

To fill these knowledge gaps, a field cable heating experiment was carried out over 4 years in southern China. *C. lanceolata* is an important tree species for timber forests in subtropical regions because of its wide cultivation area, fast growth rate, and high product quality (Bu et al., 2019). We focused on two questions: (i) how does warming affect the stoichiometric patterns of foliar C:N:P:K:Ca:Mg, non-structural carbohydrate, and stable isotope in *C. lanceolata* seedlings; and (ii) what are the effects of the stoichiometric homeostasis, non-structural carbohydrate, and stable isotope on the growth of *C. lanceolata* seedlings under the warming scenarios? Addressing these questions is critical for the future cultivation and productivity of *C. lanceolata* seedlings. Rainfall has been suggested to be the most important factor affecting the responses of foliar N and P to temperature increases (Han et al., 2011). Interestingly, during the experiment, the natural annual rainfall was higher in the early stage than in the later stage. We hypothesized the following: (i) changes in rainfall affect the responses of foliar chemical properties to warming, especially in the later stage, and exposure of *C. lanceolata* to warming increases foliar K, Ca, Mg, C:N, and non-structural carbohydrate concentrations, and water-use efficiency (H1); (ii) the stoichiometric characteristics of resource supply (especially N) do not match the demand of *C. lanceolata* seedlings under warming treatment; thus, warming leads to N non-homeostasis and affects the growth of *C. lanceolata* seedlings (H2).

## Materials and Methods

### Site Description

**Figure 1:**
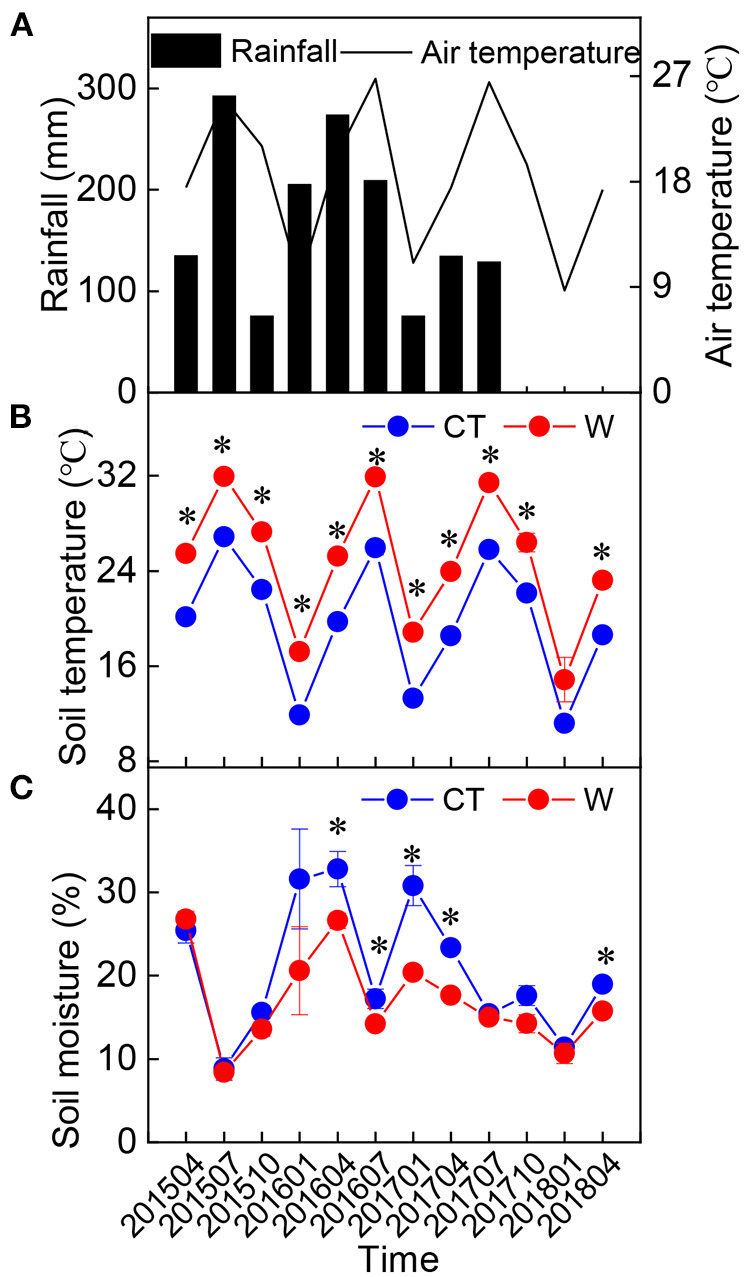
Changes in seasonal rainfall **(A)** (*n* = 1), air temperature **(A)** (*n* = 1), soil temperature **(B)** (10 cm, mean ± standard error, *n* = 5), and soil moisture **(C)** (10 cm, mean ± standard error, *n* = 5) in the study area from 2015 to 2018. *Above the dot indicates a significant difference between warming and control treatments at the level of *p* < 0.05.

The warming field experiment was conducted at the Forest Ecosystem and Global Change Research Station (26°19′ N, 117°36′ E; 300 m a.s.l.) in Sanming City, Fujian Province, China, which has a typical monsoon climate. The mean annual temperature in 2015, 2016, 2017, and 2018 was 20.1, 20.3, 20.4, and 20.3°C, respectively, based on the data obtained from a nearby weather station. The site received an annual rainfall of 1,934, 2,462, 1,142, and 1,320 mm in 2015, 2016, 2017, and 2018, respectively (Figure 1).

### Experimental Design

In 2013, 10 plots (2 m × 2 m) were established for the warming and control treatments. Each plot was separated with PVC boards inserted into soils at a depth of 0.7 m. The parent material of the soil is granite. The soil is classified as red soil according to the China soil classification systems, and is equivalent to Oxisol in the USDA Soil Taxonomy (State Soil Survey Service of China, 1998; Soil Survey Staff, 2014). On average, the soil (0–10 cm) contains 14.7 g kg^−1^ organic C and 1.4 g kg^−1^ total N; the C:N ratio was 10.5. Heating cables were used for warming and were spiral buried in soils to a depth of 10 cm. The diameter of the heating cable was 6.5 mm, the output was 17 W m^−1^ under 230 V, and the temperature increase was ~5°C, which was selected based on the RCP 8.5 scenario estimate (IPCC, 2013). To eliminate the effect of cable layout on soil disturbance, heating cables were laid in the control plots in the same way. After 5 months of adaptation, the electric power was switched on in the warming plots from March 2014. Two ECH2O-5 soil moisture sensors (Decagon, Pullman, WA, USA) and two T109 temperature sensors (Campbell Scientific Inc., Logan, UT, USA) were installed in each plot at a soil depth of 10 cm (Lin et al., 2018). The probes were buried between the cables to monitor soil moisture and temperature changes. Forty *C. lanceolata* seedlings of uniform size (25.7 ± 2.5 cm height, 3.4 ± 0.4 cm ground diameter) were selected from a nursery near the station. In November 2013, healthy *C. lanceolata* seedlings were randomly planted in plots (four seedlings per plot). The height and ground diameter of each tree was measured quarterly from April 2015 to April 2018.

### Collection of Samples and Determination of Elements

Leaves from each plot were collected quarterly from April 2015 to April 2018. All leaf samples were oven-dried at 65°C, then ground to a fine powder, and passed through a 0.25-mm sieve. Foliar C and N concentrations were determined using an elemental analyzer (Vario Max CN, Germany). After digestion with concentrated sulfuric and perchloric acids, foliar P concentration was determined using a continuous flow analyzer (Skalar, Breda, the Netherlands), and foliar K, Ca, and Mg concentrations were determined using an inductively coupled plasma optical emission spectrometer (Optima 2000 DV; Perkin Elmer, USA). Element concentration was expressed as g kg^−1^ on a dry mass basis, and the stoichiometric ratios of samples were calculated on a mass basis. Soil samples were collected from depths of 0–10 cm at three randomly selected locations using a soil core (3 cm in diameter), and samples from the same plot were combined to create a composite soil sample. Soil collection frequency was consistent with leaf sampling during 2015–2016, and soil samples were collected once a year in April during 2017–2018. All soil samples were passed through a 2-mm sieve. Soil pH was measured at a soil to water ratio of 1:2.5 (w/v) using a pH meter (Starter 300; Ohaus, Parsippany, NJ, USA). Soil organic C (SOC) and total N (TN) concentrations were determined using a CN elemental analyzer (Elementar Vario MAX, Hanau, Germany). Total P (TP) was digested with concentrated sulfuric and perchloric acids, and then analyzed using a continuous flow analyzer. Available N (including ${\text{NH}}_{4}^{+}$-N and ${\text{NO}}_{3}^{-}$-N) and available P were extracted from the soil using 2 M KCl and 0.5 M NaHCO_3_ extracting solution, respectively. The sample supernatants were then analyzed using a continuous flow analyzer. The changes in soil properties with different treatments at the different sampling times were almost consistent. The soil properties sampled in April 2015 are shown in Supplementary Table 1.

### Determination of Non-structural Carbohydrate

Approximately 100 mg of dry powdered plant sample was placed in 15-mL centrifuge tubes and incubated in 80% (v/v) ethanol at 80°C for 30 min, and then centrifuged at 21,000 *g* for 10 min. The extraction solution was used to determine soluble sugar concentration and the residue was used to determine starch concentration. Tubes with residue were left uncovered and oven-dried at 80°C, incubated in perchloric acid at 80°C for 20 min, and centrifuged at 21,000 *g* for 10 min. Glucose equivalents were used to determine soluble sugar and starch concentrations using the anthrone-sulfuric acid method (Yu et al., 2019). Starch and soluble sugar concentrations were added to calculate the total non-structural carbohydrate concentration.

### Determination of Stable Isotopic Composition

A MAT-253 isotope ratio mass spectrometer (Thermo Fisher Scientific Inc., Waltham, MA, USA) was used to measure the C and N isotope compositions. The δ notation is used to express stable isotopic abundance per mille (‰) relative to international standards. Leaf intrinsic water-use efficiency (iWUE) was calculated as follows (Farquhar et al., 1989; Huang et al., 2015):

(1) $$\begin{array}{l} iWUE=C_{a}/1.6\times \left(\delta^{13}C_{l}-\delta^{13}C_{a}+b\right)/\left(b-a\right) \end{array}$$

where, *C*_*a*_ was obtained from Mauna Loa, Hawaii; 1.6 is the ratio of gaseous diffusivity of CO_2_ to water vapor; δ^13^*C*_*l*_ is the ^13^C natural abundance in leaves; *a* is the fractionation from diffusion through stomata (= 4.4‰), and *b* is the fractionation from carboxylation by ribulose-1, 5-bisphosphate carboxylase/oxygenase (= 27‰); and δ^13^*C*_*a*_ is the ^13^C natural abundance in air calculated as follows:

(2) $$\delta^{13}C_{a}=-6.429-0.0060\text{ exp}[0.0217\left(t-1740\right)]$$

where, *t* is the year.

### Calculations

Stoichiometric homeostasis between plants and their resources was calculated as the ratio of TN_soil_ (or TP, SOC:TN, SOC:TP, and TN:TP) to N_leaf_ (or P, C:N, C:P, and N:P). A quantitative model was constructed to evaluate the response of leaf N, P, C:N, C:P, and N:P homeostasis to warming stoichiometric homeostasis (Sterner and Elser, 2002):

(3) $$\begin{array}{l} y=cx^{1/H} \end{array}$$

where, *y* is the leaf N, P, C:N, C:P, or N:P; *x* is the soil TN, TP, SOC:TN, SOC:TP, or TN:TP; *c* is a constant, and *H* is a stoichiometric homeostasis index. Accordingly, if the regression relationship was non-significant (*p* > 0.05), 1/*H* was set to 0 and the plant was considered strictly homeostatic. For all datasets with significant regressions, the degree of homeostasis was defined as follows: 0 < 1/*H* < 0.25, homeostatic; 0.25 < 1/*H* < 0.5, weakly homeostatic; 0.5 < 1/*H* < 0.75, weakly plastic; 0.75 < 1/*H*, plastic (Persson et al., 2010; Feijoó et al., 2014). This model is universal for understanding and forecasting stoichiometric equilibrium in higher plants.

To explore how plant nutrient limitation changes as a result of warming, a triaxial diagram was drawn to plot the type of nutrient limitation against the ratio of N:P:K. Dashed lines represent the critical ratios of N:P (14.5), N:K (2.1), and K:P (3.4), which divide the diagram into the following four parts: (1) N-limitation (N part); (2) P, or P and N co-limitation (P or P + N part); (3) K, or K and N co-limitation (K or K + N part); and (4) non-determinant of the type of nutrient limitation or represents non-NPK limitation (central part). For example, the opposite axis crosses the point where *K* = 0% (the horizontal axis), *N* = 59.2% (the right axis), and 10P = 40.8%, yielding a critical N:P ratio of 14.5. The point below the line represents N limitation, and the icon above the line represents P limitation or P + N co-limitation (Olde Venterink et al., 2003).

### Statistical Analyses

Prior to statistical comparison, data were log-transformed to achieve normality where needed. The stoichiometric ratios were log-transformed to ensure robust and reproducible results (Isles, 2020). Differences between the warming and control treatments were determined using independent sample *t*-tests. For the effects of warming and sampling time on foliar chemical properties, we performed an analysis of covariance (ANCOVA) using soil moisture as a covariate (function *aov* in package *multcomp*). To quantify the effect of warming on foliar chemical properties, the response ratios, that is, the ratios of foliar chemical properties with warming treatment to that with control treatment, were calculated. To evaluate the relationship between rainfall and response ratio of each foliar chemical property, partial correlation analysis was carried out using soil temperature as a covariable. Furthermore, we conducted a linear regression analysis for the relationships between rainfall and response ratios of foliar chemical properties which showed significant correlation at *p* < 0.10. The Pearson correlation analysis was used to examine the relationships between foliar chemical properties and environmental factors and the relationships among foliar chemical properties (function *corrplot* in package *corrplot*). A linear regression was performed to examine the relationships of leaf properties (N, P, C:N, C:P, and N:P) with soil properties (TN, TP, SOC:TN, SOC:TP, and TN:TP). The slopes of these linear relationships were analyzed and compared using the standardized major axis regression analysis (function *sma* in package *smatr*).

To identify how warming affects growth due to variations in soil stoichiometry, leaf stoichiometry, stoichiometric non-homeostasis, non-structural carbohydrate, and stable isotope, we performed piecewise structural equation modeling (SEM). To simplify our analysis and facilitate interpretation, a principal component analysis (PCA) was first used to reduce the number of variables, including the evaluation of soil stoichiometry, leaf stoichiometry, stoichiometric non-homeostasis, non-structural carbohydrate, stable isotope, and growth. All indexes were used as raw data for the PCA (Supplementary Table 2). We then used the result of the first principal component (PC1) (function *principal* in package *psych*) for the piecewise SEM analysis to represent soil stoichiometry, leaf stoichiometry, stoichiometric non-homeostasis, non-structural carbohydrate, stable isotope, and growth.

Simultaneously, we examined the correlations among the generated indexes. Second, we assumed that plants would exhibit stoichiometric non-homeostasis under warming conditions, which depended on leaf stoichiometry, soil stoichiometry, non-structural carbohydrate, and stable isotope, and ultimately affected their growth. In our initial model, warming was regarded as an exogenous variable; stoichiometric non-homeostasis, leaf stoichiometry, soil stoichiometry, non-structural carbohydrate, and stable isotope were treated as endogenous variables; growth was considered a response variable. Third, *d*-separation test (directional separation test), Fisher's C statistic, and Akaike information criterion (AIC) were employed to determine which paths between variables in our model should be moved. Finally, the overall goodness of model fit was evaluated using AIC (the lowest AIC indicates the best model) and Fisher's C statistic (the lowest Fisher's C value indicates the best model) (Lefcheck, 2016). We implemented piecewise SEM using the *piecewiseSEM* package (Lefcheck, 2016). The statistical analyses were performed using R 3.6.3 (R Core Team, 2019). Other analyses were carried out in SPSS 20.0 (Chicago, IL, USA). Statistical significance was set at α = 0.05. Diagrams were drawn using Origin 9.0 (OriginLab, Virginia, USA).

## Results

### Variations in Growth and Leaf Elements

**Figure 2:**
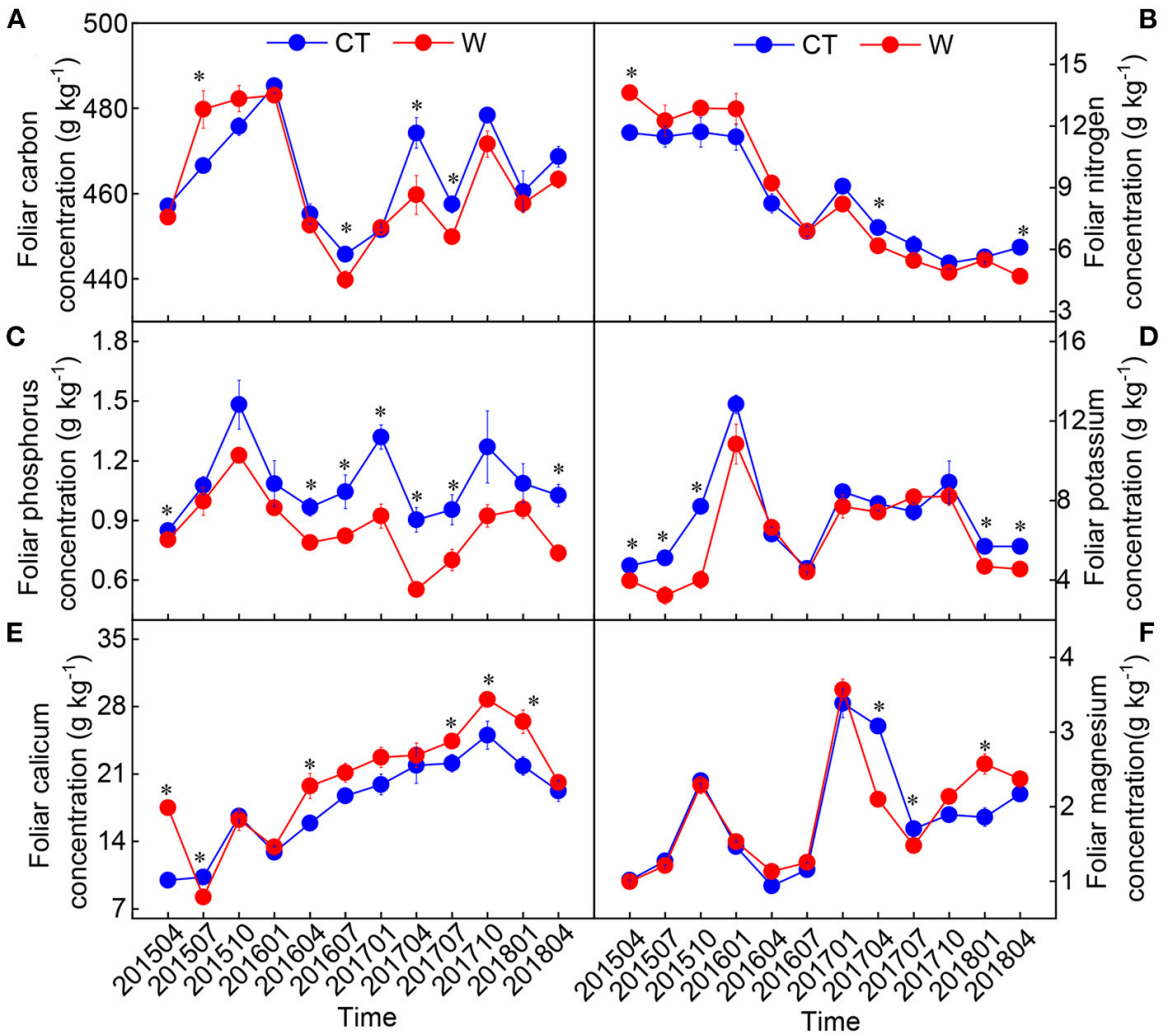
Changes in foliar elements [**(A)** carbon; **(B)** nitrogen; **(C)** phosphorus; **(D)** potassium; **(E)** calicum; **(F)** magnesium] in *Cunninghamia lanceolata* between warming (W) and control (CT) (mean ± standard error, *n* = 5). *Above the dot indicates a significant difference between warming and control treatments at the level of *p* < 0.05.

There was no significant difference in the height and ground diameter of *C. lanceolata* seedlings between the control and warming treatments (Table 1), although soil temperature was increased and soil moisture was decreased under warming treatment (Figure 1). Except for Mg, warming had a significant effect on each element in leaves (Table 2). Compared with control treatment, warming resulted in an increase in foliar N concentration by 11% in 2015 (Figure 2). Subsequently, warming decreased foliar N concentration, especially in April 2017 and April 2018 (*p* < 0.05, Figure 2). Warming had an apparent negative effect on foliar P and K concentrations. In contrast, warming increased foliar Ca concentration compared with the control (Figure 2). Significant effects of sampling time on foliar elements were observed (Table 2). With the change of time, the N concentration of leaves under different treatments gradually decreased, while the Ca concentration of leaves gradually increased (Figure 2). Foliar C, N, K, Ca, and Mg concentrations were significantly influenced by the interactive effects of warming and sampling time (Table 2).

**Table 1:** Changes in height and ground diameter of *Cunninghamia lanceolata* seedlings under warming and control treatments in April 2018 (mean ± standard error, *n* = 5).

**Index**	**Control**	**Warming**
Height (cm)	531.0 ± 12.7^a^	529.2 ± 14.5^a^
Ground diameter (mm)	82.5 ± 3.4^a^	77.1 ± 3.2^a^

*Lowercase letters in the same line denote significant differences between control and warming treatments based on a t-test at the level of p < 0.05*.

**Table 2:** Main effects of warming, time, and their interactions on foliar chemical properties using an ANCOVA with soil moisture as the covariate.

**Variables**	**W**		**T**		**W × T**	
	***F***	***p***	***F***	***p***	***F***	***p***
C	10.32	<0.01	51.27	<0.01	4.13	<0.01
N	6.59	0.01	112.62	<0.01	3.91	<0.01
P	62.99	<0.01	11.23	<0.01	1.19	0.30
K	9.03	<0.01	45.61	<0.01	3.56	<0.01
Ca	30.28	<0.01	62.80	<0.01	3.69	<0.01
Mg	0.49	0.49	143.87	<0.01	10.00	<0.01
C:N	1.49	0.22	596.56	<0.01	14.66	<0.01
C:P	37.27	<0.01	4.03	0.04	3.23	0.07
C:K	3.29	0.07	13.95	<0.01	3.15	0.08
C:Ca	8.44	<0.01	156.48	<0.01	0.01	0.99
C:Mg	0.04	0.85	44.21	<0.01	0.49	0.49
N:P	49.87	<0.01	218.07	<0.01	0.65	0.42
N:K	5.24	0.03	200.45	<0.01	11.40	<0.01
N:Ca	2.85	0.09	473.34	<0.01	3.49	0.06
N:Mg	0.11	0.74	319.49	<0.01	5.92	0.02
P:K	3.73	0.06	19.79	<0.01	6.77	0.01
P:Ca	44.74	<0.01	126.33	<0.01	1.67	0.20
P:Mg	15.84	<0.01	66.64	<0.01	3.40	0.07
Soluble sugar	0.12	0.73	154.50	<0.01	5.16	<0.01
Starch	25.61	<0.01	206.63	<0.01	6.50	<0.01
TNC	5.58	0.02	99.48	<0.01	3.33	<0.01
δ^13^C	3.27	0.07	13.46	<0.01	0.61	0.82
δ^15^N	219.09	<0.01	29.57	<0.01	3.63	<0.01
iWUE	2.01	0.16	13.16	<0.01	1.72	0.19

*W, warming effect; T, time effect; W × T, interactive effect of warming and time; TNC, total non-structural carbohydrate; iWUE, intrinsic water use efficiency*.

### Variations in Foliar Stoichiometric Ratios and Potential Nutrient Limitation

**Figure 3:**
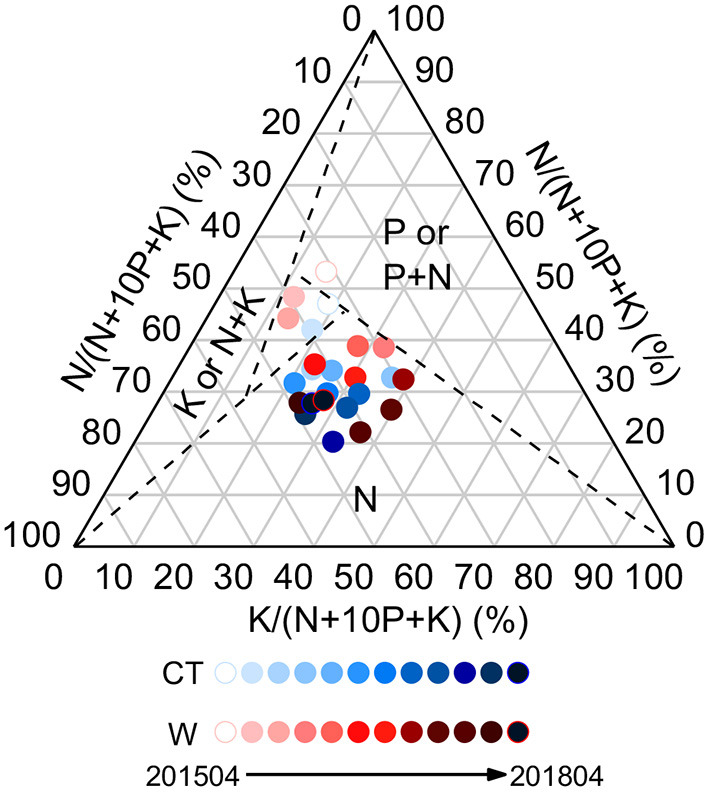
Ternary diagram of nutrient limitation in the leaves of *Cunninghamia lanceolata* under control and warming treatments.

Compared with the control, warming had a significant negative effect on foliar C:N ratio in April 2015 (*p* < 0.05), whereas the opposite effect was observed in April 2018 (Supplementary Figure 1). On average, warming increased foliar C:P and N:P ratios, while decreased foliar C:Ca, P:Ca, and P:Mg ratios (Supplementary Figure 1). The ternary diagram showed N limitation in the leaves (Figure 3).

### Variations in Leaf Non-structural Carbohydrate and Stable Isotope

**Figure 4:**
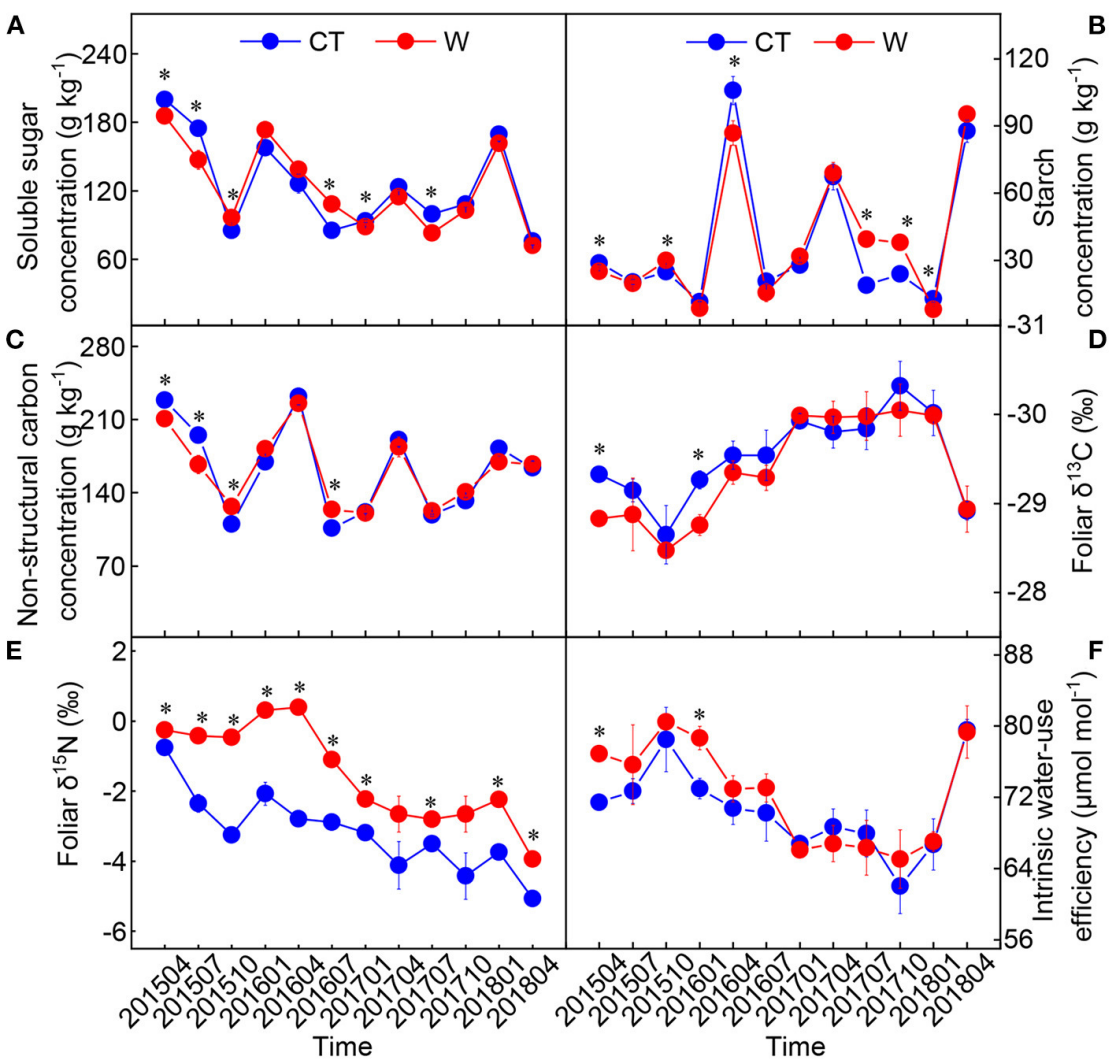
Changes in foliar soluble sugar **(A)**, starch **(B)**, total non-structure carbohydrate **(C)**, δ^13^C **(D)**, δ^15^N **(E)**, and intrinsic water use efficiency **(F)** in *Cunninghamia lanceolata* between warming (W) and control (CT) (mean ± standard error, *n* = 5). *Above the dot indicates a significant difference between warming and control treatments at the level of *p* < 0.05.

The starch, total non-structural carbohydrate, and δ^15^N were significantly influenced by warming, sampling time, and their interactive effects (Table 2). Compared with the control, warming had a negative effect on foliar δ^13^C and a positive effect on foliar iWUE from April 2015 to July 2016 (Figure 4). The value of δ^15^N in warming conditions was significantly higher than that in control conditions (*p* < 0.05, Figure 4).

### Stoichiometric Homeostasis

**Figure 5:**
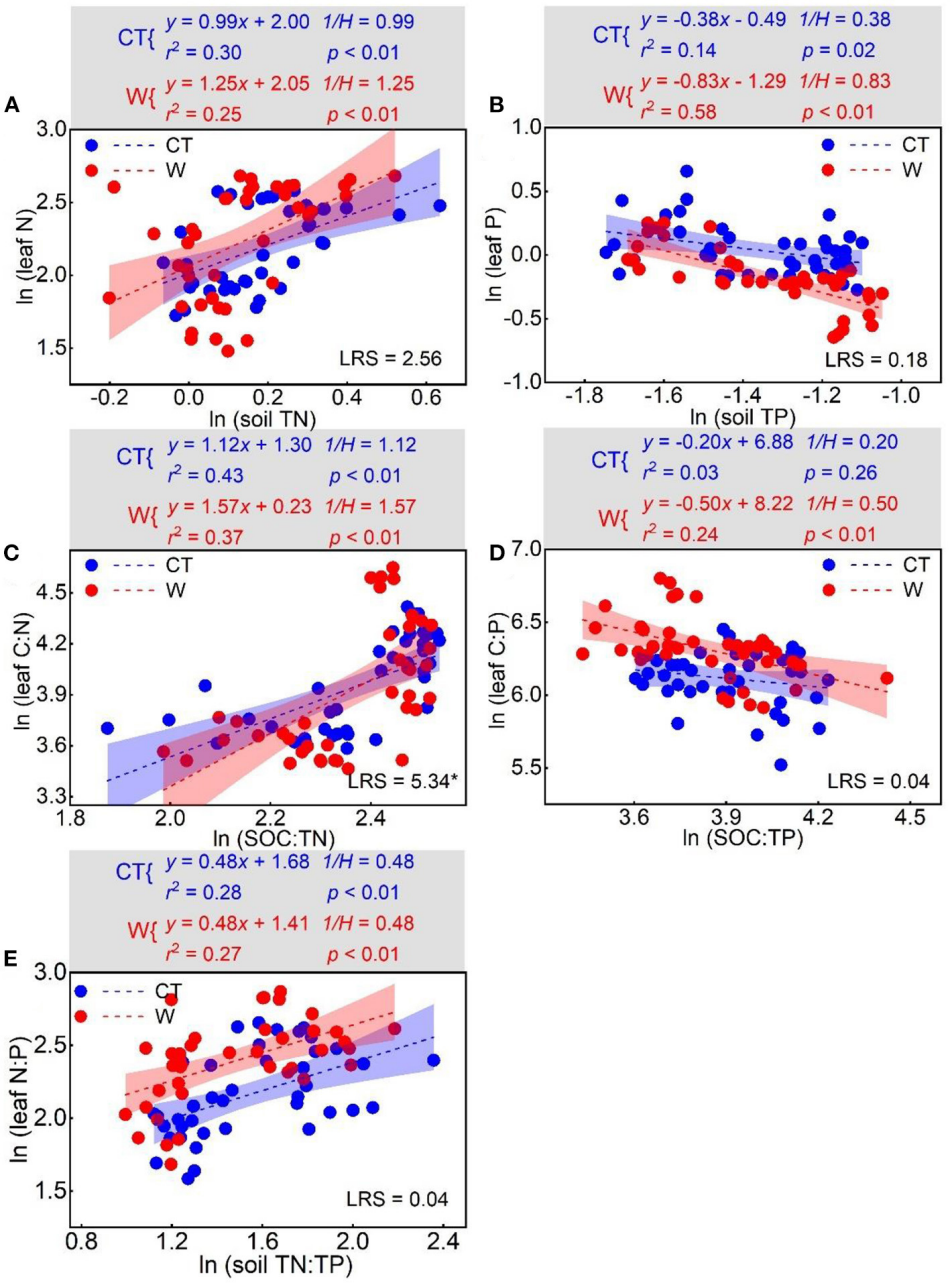
Relationships between log-transformed stoichiometries of leaf and soil resources for N homeostasis **(A)**, P homeostasis **(B)**, C:N homeostasis **(C)**, C:P homeostasis **(D)**, and N:P homeostasis **(E)** under control and warming treatments. *LRS* (likelihood ratio statistic) was the result of standardized major axis estimation for the relationships (slopes) between leaf and soil resources (control vs. warming). Significant *LRS* indicated the relationships were different at *p < 0.05.

There was a positive relationship between ln (soil TN) and ln (leaf N) (*p*_*control*_ < 0.01; *p*_*warming*_ < 0.01), between ln (SOC:TN) and ln (leaf C:N) (*p*_*control*_ < 0.01; *p*_*warming*_ < 0.01), and between ln (soil TN:TP) and ln (leaf N:P) (*p*_*control*_ < 0.01; *p*_*warming*_ < 0.01). Conversely, negative relationships between ln (soil TP) and ln (leaf P) (*p*_*control*_ = 0.02; *p*_*warming*_ < 0.01), and between ln (soil SOC:TP) and ln (leaf C:P) (*p*_*control*_ = 0.26; *p*_*warming*_ < 0.01) were observed. Under control conditions, the *H* values for N, P, C:N, C:P, and N:P were 1.01, 2.63, 0.89, 5.00, and 2.08, respectively. Under warming conditions, the *H* values for N, P, C:N, C:P, and N:P were 0.80, 1.20, 0.64, 2.00, and 2.08, respectively. There was a significant difference in the *H* value for C:N (*p* = 0.02) between the control and warming treatments (Figure 5).

### Integrated Adaptive Mechanisms of Stoichiometry to Growth

**Figure 6:**
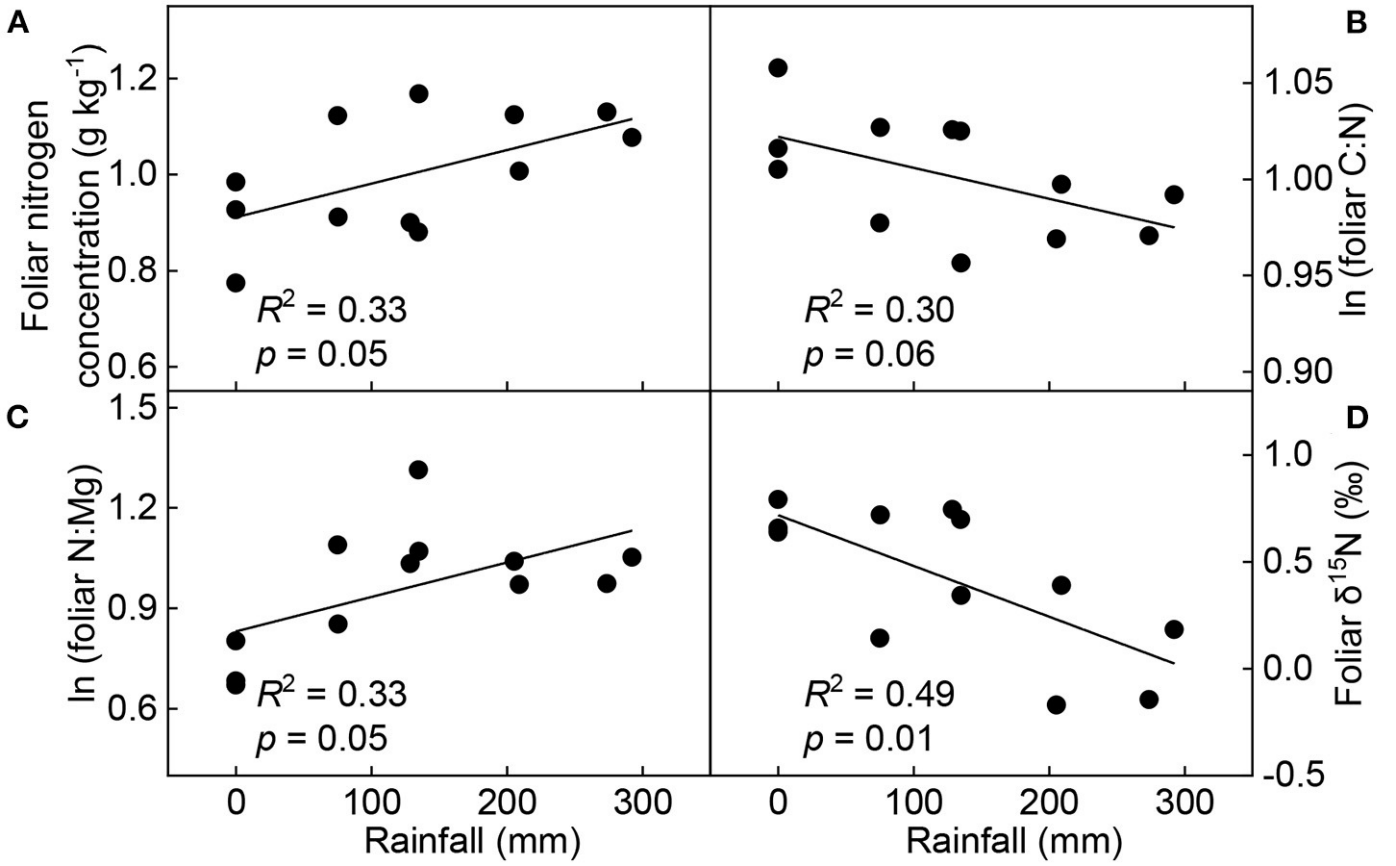
Linear relationships between seasonal rainfall and response ratios of foliar N concentration **(A)**, C:N ratio **(B)**, N:Mg ratio **(C)**, and δ^15^N **(D)** (*n* = 12). The response ratios were calculated as the ratios of foliar chemical properties under warming treatment to those under control treatment.

The Pearson correlation analysis revealed that soil temperature negatively correlated with soil moisture (*p* < 0.01), the concentrations of P (*p* = 0.01), K (*p* < 0.01), Mg (*p* < 0.01), soluble sugar (*p* = 0.01), and total non-structural carbohydrate (*p* = 0.02) in leaves, respectively. However, it significantly positively correlated with foliar C:P (*p* = 0.01), C:K (*p* < 0.01), C:Mg (*p* < 0.01), N:P (*p* = 0.04), N:K (*p* < 0.01), N:Mg (*p* = 0.02), P:K (*p* < 0.01), and P:Mg (*p* = 0.04) ratios, and δ^15^N (*p* < 0.01; Supplementary Figure 2). Taking into account the changes in soil temperature (Supplementary Table 3), we found that rainfall was positively correlated with the response ratios of foliar N concentration (*p* = 0.05) and N:Mg (*p* = 0.05), and negatively correlated with the response ratios of C:N (*p* = 0.06) and δ^15^N (*p* < 0.01; Figure 6).

**Figure 7:**
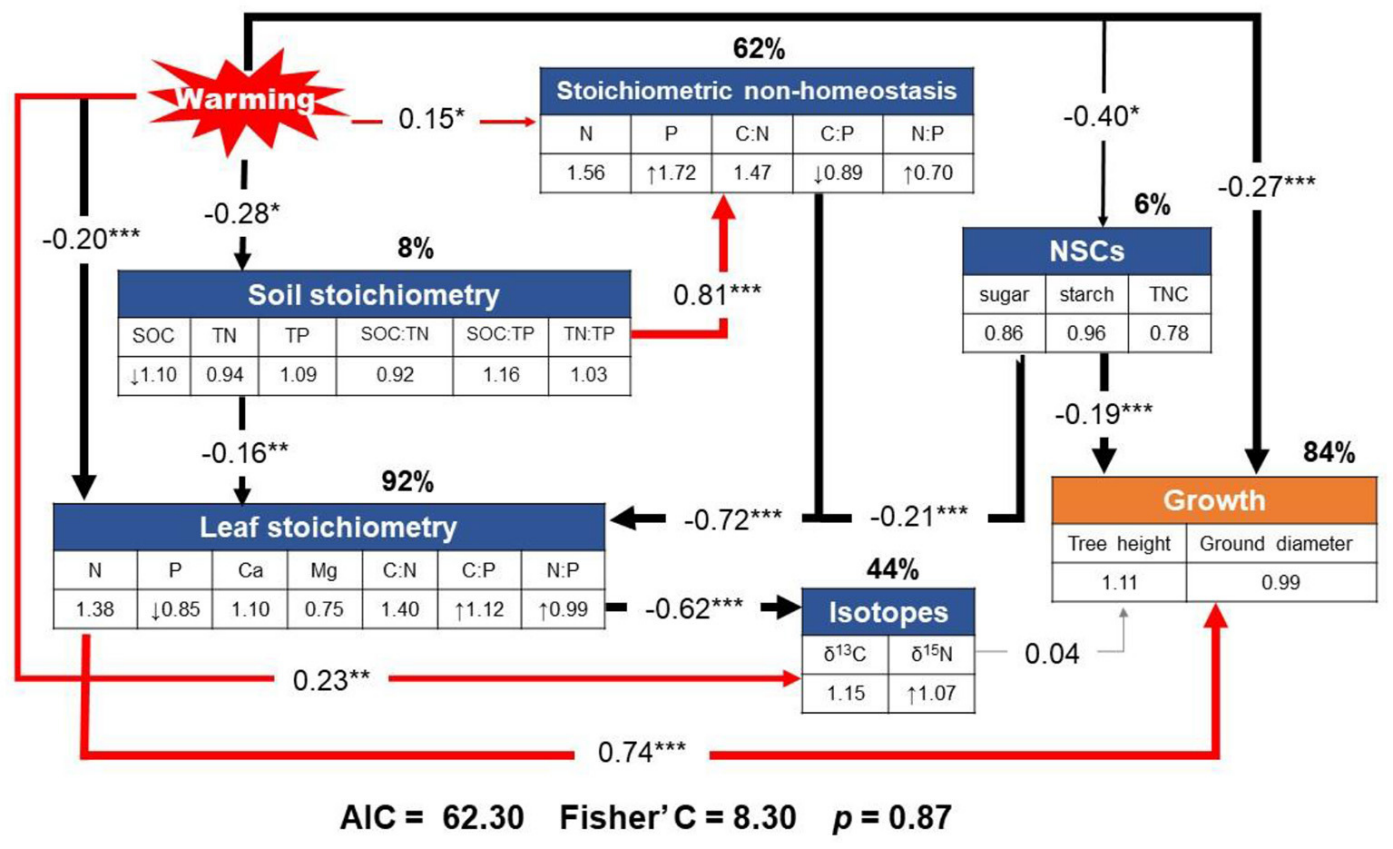
Results from the final piecewise structural equation model illustrating the effects of changes in stoichiometric non-homeostasis due to warming on plant nutrients and the indirect effects of stoichiometric non-homeostasis on growth via pathways through leaf stoichiometry (i.e., nutrient-use efficiency), non-structural carbohydrates (NSCs), and stable isotopes (i.e., δ^15^N, as a proxy for N cycling). Path coefficients (correlation coefficients) along the arrows are standardized by the mean of each index. The symbols “↑” and “↓” indicate a significant increase and decrease, respectively, in response to warming. The number in the glen check denotes the slope of the linear model of each parameter with warming as a continuous predictor. Red and black arrows represent significant positive and negative relationships (*p* < 0.05), respectively. Gray arrow represents insignificant correlation. *, **, and *** denote statistical significance at *p* < 0.05, *p* < 0.01, and *p* < 0.001, respectively. Percentages close to variables present the variance explained by the model (*R*^2^).

Our piecewise SEM model (Fisher's C = 8.30, *p* = 0.87, indicating adequate fit) showed that warming directly and indirectly affected stoichiometric non-homeostasis, together with leaf stoichiometry, soil stoichiometry, non-structural carbohydrate, and stable isotope, which explained 84% of the total variance in growth (Figure 7).

## Discussion

### Response of Growth to Warming

Due to the limitation of mountainous terrain and the influence of a subtropical monsoon climate, electric heating cable was used to increase soil temperatures in the present study. Compared with other warming methods, the use of heating cable produced higher and more accurate warming magnitudes (Chen et al., 2015). Subsequent changes in soil resource availability may lead to a faster response of root biomass (Gao et al., 2021), but the effect of soil warming on aboveground growth may be insignificant in the short term or lagging (Weldearegay et al., 2012; Li et al., 2016). This could explain why warming had no significant effect on tree height and ground diameter (Table 1).

### Response of Stoichiometry to Warming

Previous research indicates that foliar N concentration increases with temperature (Yue et al., 2019; Yu et al., 2019; Litton et al., 2020), because warming increases the rate of soil organic matter mineralization and soil N availability (Bai et al., 2013). The biogeochemical hypothesis suggests that the increase of foliar N under warming conditions is associated with the increases of plant physiological and photosynthetic activities and the increase of plant N uptake capacity (Reich and Oleksyn, 2004). Our analysis of leaves sampled in 2015 and 2016 supports these previous observations. After that, warming had a negative effect on foliar N concentration (Figure 2). This conflicting result is consistent with our hypothesis (H1). Variations in seasonal rainfall were observed during the experiment. We found that the response ratio of foliar N to warming was closely correlated with rainfall (Figure 6 and Supplementary Table 3). Accordingly, compared to 2015 and 2016, a relative decrease of rainfall in 2017 and 2018 might have exacerbated warming-induced drought stress. Such a decrease in foliar N concentration due to warming or drought has been observed in other ecosystems (Sardans et al., 2008a; Gargallo-Garriga et al., 2015).

Plants may invest fewer nutrients in protein production to sustain biochemical reactions under warming conditions (Reich and Oleksyn, 2004). Thus, foliar P concentration decreases as average temperatures increase. Consistent with these findings, we documented a strong and negative response of foliar P concentration to warming (Figure 2). As a result, warming increased foliar C:P and N:P ratios and decreased foliar P:Ca and P:Mg ratios (Supplementary Figure 1). A meta-analysis of data from 76 field warming studies found that warming increases plant N:P ratio overall (Yue et al., 2017). Another meta-analysis conducted by Tian et al. (2019) also suggested that warming decreases foliar P:Ca and P:Mg ratios. Overall, these results showed that warming increases the P-use efficiency of leaves.

Foliar K, Ca, and Mg are important indicators of plant water deficit (Sardans and Peñuelas, 2015; Wu et al., 2018; Prieto and Querejeta, 2020). We hypothesized that warming would increase foliar K, Ca, and Mg concentrations (H1), but our findings only support that warming increases foliar Ca concentration (Figure 2). This result suggests that foliar Ca play an important role in regulating foliar osmotic pressure. In addition, there was a strong negative relationship between foliar N and Ca (or Mg) concentrations (Supplementary Figure 2); this shows that higher N levels are required for osmosis, enzyme activation, and electrochemical processes. Evidently, under warming conditions, a decrease in N concentration in the later stage and an increase in Ca concentration will gradually affect the integrity and chemical stability of plant cell walls and cell membranes. This may be one of the reasons that the aboveground growth did not change significantly under warming conditions.

### Responses of Non-structural Carbohydrate and Intrinsic Water-use Efficiency to Warming

Seasonal dynamics of non-structural carbohydrate can reflect the proportion of C source to C sink at different developmental stages (Yu et al., 2019). Higher temperatures have been found to reduce the non-structural carbohydrate concentration in many trees, such as *Picea mariana* (Way and Sage, 2008), *Eucalyptus globulus* (Duan et al., 2013), and *Abies faxoniana* (Yu et al., 2019). Here, the concentration of non-structural carbohydrate decreased from April to July (Figure 4). This can be explained by the increase in the leaf respiration rate due to seasonal temperature increases, which leads to an increase in photoassimilate consumption (Herrera-Ramirez et al., 2020; Maxwell et al., 2020). Strong negative relationship between growth rate and starch concentration in *C. lanceolata* supports this conclusion (*y*_treeheight_ = −0.76*x*_starch_ + 57.31, *R*^2^ = 0.21, *p* < 0.01 for tree height growth rate; *y*_grounddiameter_ = −1.27*x*_starch_ + 63.02, *R*^2^ = 0.25, *p* < 0.01 for ground diameter growth rate). Together with the changes in foliar P, C:P, and P:K, these results can be attributed to a higher growth rate from April to July. During this period, the cotyledons were fully developed and presented a new branch that consumes more photoassimilates than those fixed. Contrarily, we found that foliar non-structural carbohydrate increased from October (fall) to January (winter) (Figure 4), which may have protected the plant from intra- and intercellular freezing (Tixier et al., 2020).

The intrinsic water-use efficiency of plants refers to the amount of C required for the evaporation of water, reflecting the transaction cost between plant C assimilation and water loss (Peng et al., 2020; Belmecheri et al., 2021). A positive effect of warming on intrinsic water-use efficiency was observed in the years with higher annual rainfall (Figure 4), indicating that warming considerably influences the water regulation function of *C. lanceolata* seedlings. That is, warming increased C fixation in *C. lanceolata* seedlings and reduced the water consumption cost of fixing a unit of C. Interestingly, despite a decrease in annual rainfall in the later periods, warming had no significant effect on the intrinsic water-use efficiency. This suggests that there is a counterbalancing effect between plant C assimilation and water loss after warming as the seedlings develop. This strategy may help young forests to successfully survive in future climate change scenarios.

### Mechanisms of Stoichiometry to Growth Under Warming

In line with our second hypothesis, there was non-homeostasis of N and C:N under warming conditions (Figure 5). To understand what caused N and C:N to be non-homeostatic, three phenomena were explored. First, rainfall variability affects the response of N to warming. Specifically, the effect of warming on the foliar C:N ratio changed from negative to positive (Supplementary Figure 1). In 2017 and 2018, when there was less rainfall, warming resulted in an increased foliar C:N ratio, which was likely the consequence of warming-induced lower foliar N concentration. Increases in the foliar C:N ratio under warming have been interpreted as helping to protect leaves from a water deficit (Gargallo-Garriga et al., 2015; Viciedo et al., 2019; Wedow et al., 2019). In association with the changes of Ca and Mg concentrations, plant growth, and the temperature-plant physiological hypothesis (Reich and Oleksyn, 2004), there is evidence that the decrease in foliar N concentration under warming conditions is related to the mechanism of acclimation. Furthermore, it is likely that warming may cause thermal stress that alters the N cycle.

Second, warming increases N loss. To further explore this, we determined plant δ^15^N that may act as a proxy for the available N changes in soil (Hobbie and Högberg, 2012; Piao et al., 2020). We found that warming significantly increased foliar δ^15^N (Figure 4), indicating warming increases the fractionation of δ^15^N and exacerbates the loss of lighter ^14^N in the soil. Likewise, warming treatments triggered large N losses in desert steppes and subarctic ecosystems (Maranon-Jimenez et al., 2019; Ren et al., 2021). Moreover, δ^15^N fractionation was intensified by lower rainfall, as evidenced by the significant negative correlation between seasonal rainfall and the response ratio of foliar δ^15^N to warming. So, what happens when warming increases N loss?

Third, plant growth is limited by N. That's the answer to the question above. According to the N:P threshold proposed by Koerselman and Meuleman (1996), plant growth may be limited by N when foliar N:P is <2.64 (ln 14 = 2.64) and N concentration is <20 g kg^−1^. On the contrary, plant growth may be limited by P when foliar N:P is >2.77 (ln 16 = 2.77) and P concentration is <1 g kg^−1^. In the present study, the average foliar N:P was 2.02 and 2.23 under control and warming treatments, respectively, and foliar N concentration was <20 g kg^−1^. These findings indicate that N could limit the productivity of *C. lanceolata* seedlings, which can also be confirmed by the ternary diagram (Figure 3). Although previous studies have shown that P limitation occurs in subtropical forest ecosystems, and the result of N limitation seems conflicting with higher P-use efficiency, this can be reconciled by the multiple limitation hypothesis (Hou et al., 2020). Additionally, the value of N:P stoichiometric homeostasis was identical between the treatments, implying a balance of N and P limitation under warming conditions.

The results of the SEM showed that warming indirectly affected growth by altering stoichiometry (Fisher's C = 8.30, *p* = 0.87; Figure 7). The non-significant change in tree growth following warming was directly linked to leaf stoichiometry, soil stoichiometry, non-structural carbohydrate, and stable isotope, accompanied by stoichiometric non-homeostasis accounting for 84% of the variation in the growth of *C. lanceolata*. Among them, the higher degree of stoichiometric non-homeostasis (62%) with warming may have contributed to the lack of change in growth. These results suggest that higher homeostatic regulation capacity will play an important role in biomass production under future climate conditions.

## Conclusions

In summary, soil warming has significant effects on most of the foliar chemical properties, but has no effect on the growth of *C. lanceolata* seedlings. The responses of foliar N, C:N, and δ^15^N to soil warming were related to rainfall changes. Under warming conditions, the growth of *C. lanceolata* seedlings showed a co-limitation of N and P. The stoichiometric homeostasis model revealed that warming increases the degree of non-homeostasis of N and C:N. There are three reasons for this: interannual variations in rainfall, increased losses of N, and N limitation in leaves. In contrast, *C. lanceolata* seedlings showed higher homeostasis of N:P. The stoichiometric homeostasis values of N:P were consistent between the control and warming treatments, indicating that the limitation of N and P was balanced. We combined plant nutrient adaptive mechanisms related to stoichiometry with plant growth using the piecewise SEM analysis, and found that the non-significant change in growth of *C. lanceolata* seedlings following soil warming was indirectly linked to foliar stoichiometric homeostasis. Although the results obtained for seedlings under warming conditions should be carefully extrapolated to adult trees, our findings indicate that the changes in soil temperature might not substantially influence *C. lanceolata* growth.

## Data Availability Statement

Publicly available datasets were analyzed in this study. This data can be found here: https://doi.org/10.6084/m9.figshare.12951359.

## Author Contributions

QZ: conceptualization, writing—original draft preparation, investigation, and software. DL: software. LY, JZ, and XL: investigation. JX, ZY, and DX: supervision and resources. YC and YY: conceptualization, validation, writing—reviewing and editing, and funding acquisition. All authors contributed to the article and approved the submitted version.

## Conflict of Interest

The authors declare that the research was conducted in the absence of any commercial or financial relationships that could be construed as a potential conflict of interest.

## Publisher's Note

All claims expressed in this article are solely those of the authors and do not necessarily represent those of their affiliated organizations, or those of the publisher, the editors and the reviewers. Any product that may be evaluated in this article, or claim that may be made by its manufacturer, is not guaranteed or endorsed by the publisher.
