# Supplementary material for: Variations in Rainfall Affect the Responses of Foliar Chemical Properties of Cunninghamia lanceolata Seedlings to Soil Warming
Source: Front Plant Sci. 2021 Jul 30;12:705861. doi: 10.3389/fpls.2021.705861 (PMC8363246; doi:10.3389/fpls.2021.705861)
Supplement: Supplementary file 1 [file Presentation_1.pdf]

Supplementary Material

Supplementary Table 1. Soil chemical analysis of samples collected in April 2015 (mean ± standard error, n = 5). Lowercase letters denote significant differences between control and warming treatments based on a *t*-test at the level of $p < 0.05$. SOC, soil organic carbon; TN, soil total nitrogen; TP, soil total phosphorus; AN, available nitrogen; AP, available phosphorus.

Treatments	pH	SOC (g kg^{-1})	TN (g kg^{-1})	TP (g kg^{-1})	AN (mg kg^{-1})	AP (mg kg^{-1})
Control	4.7 ± 0.06 a	14.3 ± 0.45 a	1.4 ± 0.05 a	0.3 ± 0.01 a	8.4 ± 0.08 b	1.6 ± 0.14 a
Warming	4.6 ± 0.12 a	12.7 ± 1.00 a	1.2 ± 0.11 a	0.3 ± 0.01 a	12.9 ± 1.35 a	1.9 ± 0.10 a

Supplementary Table 2. Results of principal component analysis of variables in the following categories: stoichiometric non-homeostasis, leaf stoichiometry, soil stoichiometry, non-structural carbohydrates, and stable isotopes.

Variables	Component 1
**Stoichiometric non-homeostasis**	
N stoichiometric non-homeostasis	−0.07
P stoichiometric non-homeostasis	−0.79
C:N stoichiometric non-homeostasis	−0.08
C:P stoichiometric non-homeostasis	0.87
N:P stoichiometric non-homeostasis	0.97
Cumulative (%)	76.29
**Leaf stoichiometry**	
Leaf N	−0.97
Leaf P	−0.34
Leaf Ca	0.73
Leaf Mg	0.51
Leaf C:N	0.97
Leaf C:P	0.34
Leaf N:P	−0.78
Cumulative (%)	62.46
**Non-structural carbohydrate**	
sugar	0.43
starch	−0.99
Total non-structural carbohydrate	−0.36
Cumulative (%)	81.74
**Stable isotopes**	
$\delta^{13}C$	−0.26
$\delta^{15}N$	−0.99
Cumulative (%)	90.80
**Soil stoichiometry**	
SOC	0.06
TN	0.86
TP	−0.89
SOC:TN	−0.84
SOC:TP	0.95
TN:TP	0.99
Cumulative (%)	82.00
**Growth**	
Tree height	−0.99
Ground diameter	−0.99
Cumulative (%)	98.32

Supplementary Table 3. Partial correlations between seasonal rainfall and response ratios of soil moisture and foliar chemical properties after controlling soil temperature. Zero-order means there is no controlling factor. The response ratios were calculated as the ratios of foliar chemical properties under the warming treatment to those under the control treatment. TNC, total non-structural carbohydrate, iWUE, intrinsic water use efficiency. † and * denote statistical significance at $p < 0.01$ and $p < 0.05$, respectively.

Variable	Rainfall	Rainfall
	Zero-order	Soil temperature (control)
Soil moisture	0.13	0.18
C	0.32	0.32
N	0.58†	0.58†
P	0.31	0.31
K	0.08	0.08
Ca	−0.14	−0.15
Mg	−0.21	−0.22
C:N	−0.55†	−0.55†
C:P	−0.35	−0.35
C:K	−0.03	−0.03
C:Ca	0.26	0.26
C:Mg	0.26	0.27
N:P	−0.01	0.01
N:K	−0.36	−0.37
N:Ca	−0.09	−0.10
N:Mg	0.57†	0.58†
P:K	−0.13	−0.14
P:Ca	−0.38	−0.39
P:Mg	−0.34	−0.39
Soluble sugar	0.24	0.24
Starch	−0.28	−0.31
TNC	−0.17	−0.17
$\delta^{13}C$	−0.38	−0.38
$\delta^{15}N$	−0.70*	−0.71*
iWUE	0.29	0.29

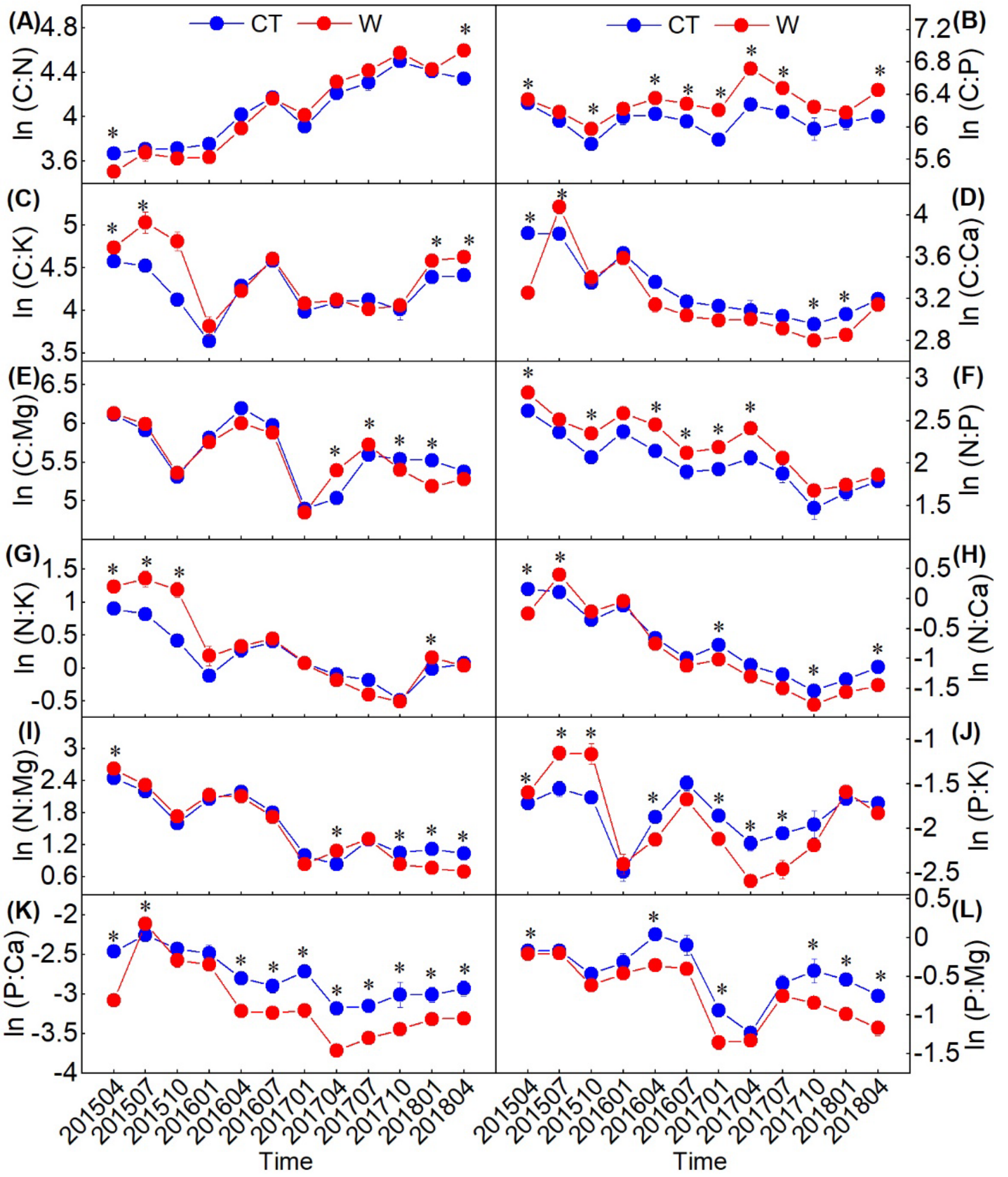

Supplementary Figure 1. Changes in foliar stoichiometric ratios (**A**, C:N; **B**, C:P; **C**, C:K; **D**, C:Ca; **E**, C:Mg; **F**, N:P; **G**, N:K; **H**, N:Ca; **I**, N:Mg; **J**, P:K; **K**, P:Ca; **L**, P:Mg) in *Cunninghamia lanceolata* seedlings between warming (W) and control (CT) treatments (mean ± standard error, n = 5). * above the dot indicates a significant difference between warming and control treatments at the level of $p < 0.05$.

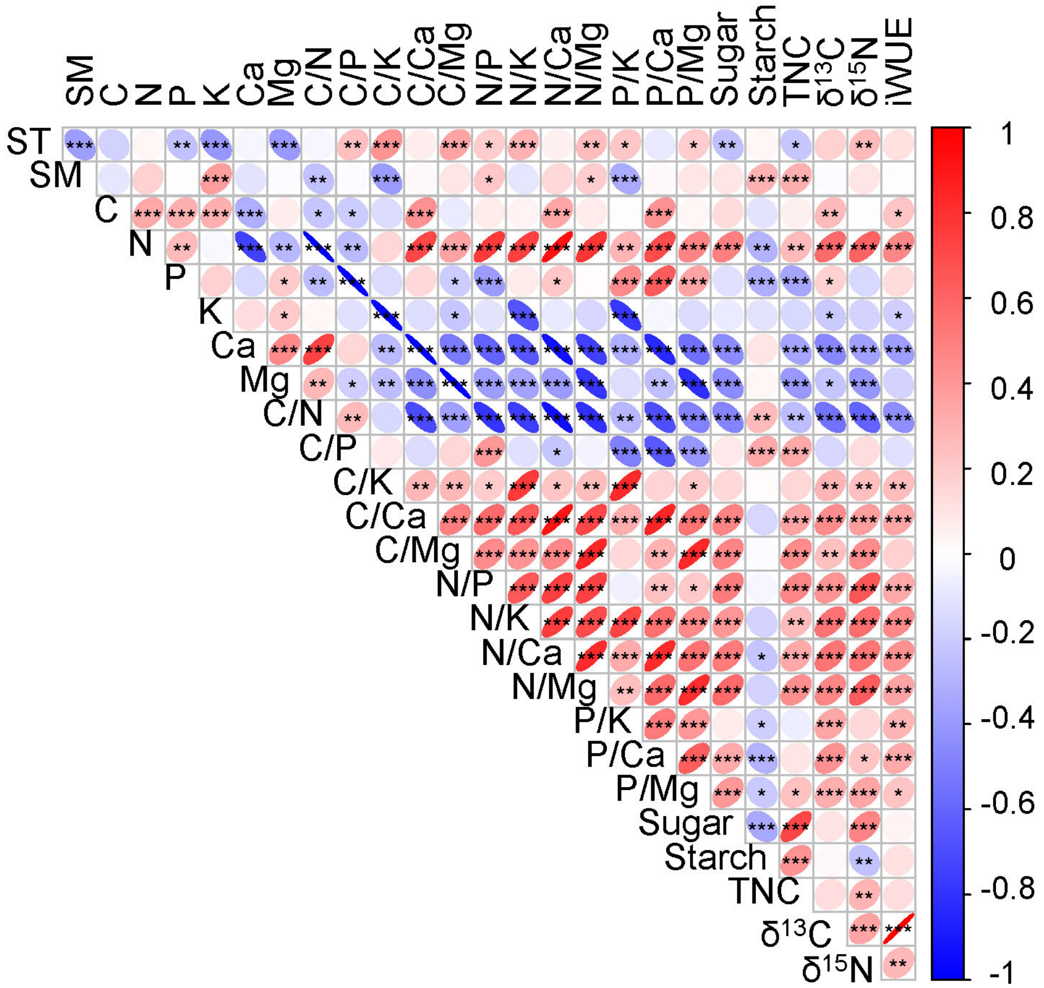

Supplementary Figure 2. Pearson's correlations among response variables in *Cunninghamia lanceolata* seedlings during 2015−2018 (n = 120). ST, soil temperature; SM, soil moisture; TNC, total non-structural carbohydrate; iWUE, intrinsic water use efficiency. *, **, and *** denote statistical significance at $p < 0.05$, $p < 0.01$, and $p < 0.001$, respectively.
